# Implementation of prediction models in the emergency department from an implementation science perspective—Determinants, outcomes and real-world impact: A scoping review protocol

**DOI:** 10.1371/journal.pone.0267965

**Published:** 2022-05-12

**Authors:** Sze Ling Chan, Jin Wee Lee, Marcus Eng Hock Ong, Fahad Javaid Siddiqui, Nicholas Graves, Andrew Fu Wah Ho, Nan Liu

**Affiliations:** 1 Health Services Research Centre, Singapore Health Services, Singapore, Singapore; 2 Programme in Health Services and Systems Research, Duke-NUS Medical School, Singapore, Singapore; 3 Centre for Quantitative Medicine, Duke-NUS Medical School, Singapore, Singapore; 4 Department of Emergency Medicine, Singapore General Hospital, Singapore, Singapore; 5 Prehospital Emergency Research Centre, Duke-NUS Medical School, Singapore, Singapore; 6 SingHealth AI Health Program, Singapore Health Services, Singapore, Singapore; 7 Institute of Data Science, National University of Singapore, Singapore, Singapore; Northumbria University, UNITED KINGDOM

## Abstract

The number of prediction models developed for use in emergency departments (EDs) have been increasing in recent years to complement traditional triage systems. However, most of these models have only reached the development or validation phase, and few have been implemented in clinical practice. There is a gap in knowledge on the real-world performance of prediction models in the ED and how they can be implemented successfully into routine practice. Existing reviews of prediction models in the ED have also mainly focused on model development and validation. The aim of this scoping review is to summarize the current landscape and understanding of implementation of predictions models in the ED. This scoping review follows the Systematic reviews and Meta-Analyses extension for Scoping Reviews (PRISMA-ScR) checklist. We will include studies that report implementation outcomes and/or contextual determinants according to the RE-AIM/PRISM framework for prediction models used in EDs. We will include outcomes or contextual determinants studied at any point of time in the implementation process except for effectiveness, where only post-implementation results will be included. Conference abstracts, theses and dissertations, letters to editors, commentaries, non-research documents and non-English full-text articles will be excluded. Four databases (MEDLINE (through PubMed), Embase, Scopus and CINAHL) will be searched from their inception using a combination of search terms related to the population, intervention and outcomes. Two reviewers will independently screen articles for inclusion and any discrepancy resolved with a third reviewer. Results from included studies will be summarized narratively according to the RE-AIM/PRISM outcomes and domains. Where appropriate, a simple descriptive summary of quantitative outcomes may be performed.

## Introduction

Patients presenting to the emergency department (ED) have varying needs for urgent medical attention and limited hospital resources often necessitate prioritization of some patients over others [1]. Overcrowding of the ED is a common and increasing problem and can lead to adverse patient outcomes [2]. EDs therefore need to quickly determine the urgency and level of care required for each patient in order to optimize the allocation of scarce hospital resources [3]. To achieve this, most modern EDs have a triage process to assess patients’ severity of illness or injury upon arrival, assign priorities and then provide appropriate treatment [3,4]. Currently, ED triage is most commonly guided by semi-subjective scale-based systems, with some notable examples being the Emergency Severity Index (ESI) [5] and the Canadian Triage and Acuity Scale (CTAS) [6]. Using a mixture of qualitative and quantitative metrics, these scale-based protocols guide the healthcare practitioner in assigning the patient to a label that reflects his/her required level of care. Although scale-based systems have been widely implemented and have shown their usefulness, their accuracy is highly dependent on the triaging doctors’ or nurses’ experience [3]. In recent years, various prediction models have been developed for ED patients that could complement subjective scale-based triage processes and further optimize management of patients in the ED [7,8]. These models are typically derived from real-world data and utilize a range of statistical and machine learning tools, ranging from traditional regression models to cutting-edge neural networks [9]. Some examples include models predicting in-hospital mortality [10], intensive care unit (ICU) admission or readmission [11–13]. However, among the many models developed, few were externally validated and even fewer had their impact on clinical practice analysed [7].

Nevertheless, some of these prediction models may have been implemented into routine clinical practice with the increasing emphasis on harnessing big data and building learning healthcare systems [14]. While the area under curve (AUC) and other quantitative summary statistics are used in model development and validation, they do not entirely capture the actual consequences of model implementation [15]. Predictive analytics promise to improve patient outcomes but several intervening steps leading to providers responding appropriately to model outputs are necessary to result in actual patient benefit [16]. Studying the implementation process and its impact on outcomes can identify potential barriers and facilitators to implementation and strategies that may promote implementation [17].

There have been systematic reviews providing informative overviews of prediction models in the ED primarily in terms of model structure, development, and performance [7,12,18]. One review on clinical decision support systems for triage in ED found that less than half of the included studies had an implementation phase even though the majority of them showed promising potential in the validation phase [7]. These findings suggest that there exists a host of barriers which are unrelated to performance and that despite the paucity in implementation, these barriers can be overcome with proper knowledge and execution. Furthermore, to the best of our knowledge, there has been no review of the process and outcomes of implementing these models into routine clinical practice. Therefore, a gap still exists in our current understanding of the logistical and administrative challenges involved in prediction model implementation in the ED. This gulf in the current body of knowledge also extends to an understanding of how outcomes of such implemented models are assessed and perceived by the healthcare providers. This points to a need for a scoping review on this topic, a type of systematic evidence synthesis where the intent is to assess and understand the extent of knowledge or map the concepts in a particular field, rather than to answer a specific clinical question to aid decision making as is the case for a systematic review [19].

**Fig 1:**
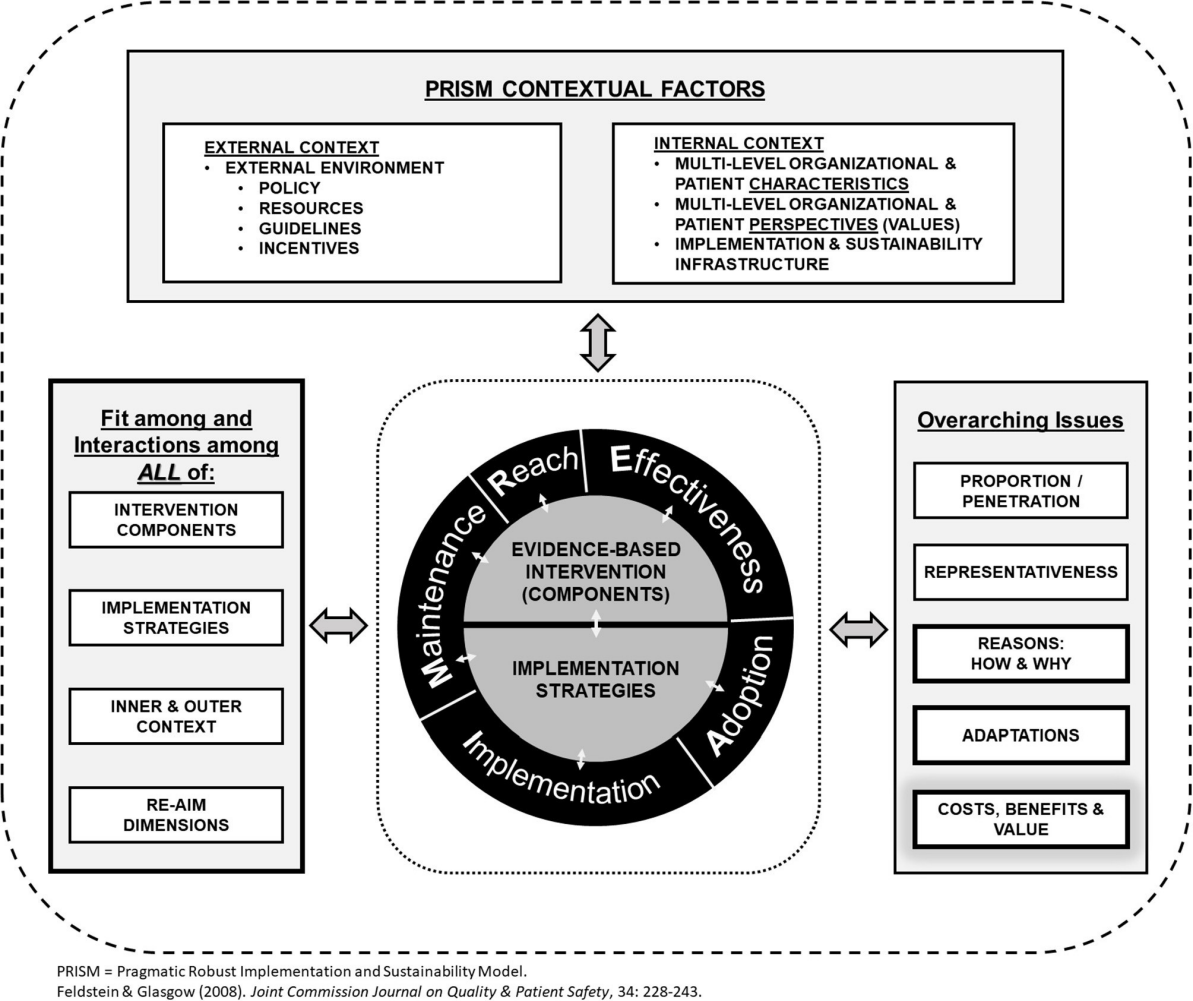
The revised RE-AIM/PRISM framework [20].

To understand both the contextual determinants as well as outcomes affecting implementation success into routine clinical practice, we will be using the revised, enhanced Reach, Effectiveness, Adoption, Implementation, Maintenance (RE-AIM)/Practical, Robust, Implementation, and Sustainability Model (PRISM) 2019 framework to guide this review (Fig 1) [20]. The revised RE-AIM/PRISM framework retains the original 5 RE-AIM domains, which can be used as evaluation outcomes for an implementation effort and includes multilevel contextual determinants from PRISM that can explain these implementation outcomes [20]. This would be useful in guiding future implementation studies, but also in providing valuable insight into the factors that influence to the success of implementing prediction models in the ED [21,22].

This review is also focused on implementation of prediction models into routine ED practice. Implementation is a process, where implementation studies can occur from pre-implementation to the maintenance phase. Studies focused on implementation outcomes at any stage are therefore relevant for our review. Prediction models typically progress from development, internal validation, external validation to impact assessment before being implemented [23,24]. Model performance prior to routine implementation is therefore theoretical, even if performed on actual patient data. For effectiveness, we therefore chose to only include results after implementation, as this represents ‘real-world effectiveness’.

The aim of this scoping review is to summarize the current landscape and understanding of implementation of prediction models in the ED from an implementation science angle. Specifically, apart from a descriptive summary of characteristics of prediction models implemented in clinical practice, we will summarize the implementation outcomes and contextual determinants affecting implementation success according to the revised RE-AIM/PRISM framework [20].

## Methods

This scoping reviews follows the Systematic reviews and Meta-Analyses extension for Scoping Reviews (PRISMA-ScR) checklist [25]. At the time of writing, we have completed the searching, title and abstract screening, and full-text screening.

### Inclusion and exclusion criteria

We expected that reports on prediction model implemented in the ED would have varying degrees of focus on implementation outcomes and reporting of implementation strategies. We also recognized that not all implementation studies may be comparative. For clarity, we have combined the PICOT (Population, Intervention, Comparison, Outcome and Time) framework and the Standards for Reporting Implementation Studies (StaRI) guidelines [26] to differentiate the intervention and implementation strategy of interest. The intervention describes the type of prediction models of focus in this review, and implementation strategy describes the studies. We therefore included studies that satisfied the PICOT elements for both the intervention and implementation strategy (Table 1). For the implementation strategy, the intervention and comparison elements were seen as optional to accommodate non-comparative studies.

**Table 1:** Inclusion and exclusion criteria.

PICOT elements	Implementation strategy	Intervention
Population	Any relevant stakeholder in the implementation of the intervention (e.g., ED staff, patients, management, etc)	Patients admitted to any hospital or healthcare facility based EDs, including any specific subset of these patients
Intervention	If available: any actions to promote successful implementation of the intervention	Any prediction model/score/algorithm containing at least 1 predictor and a numeric output (e.g., score, points, probability, etc)
Comparison	If available: no strategy or an alternative strategy	Usual practice (without or before use of the prediction model)
Outcomes	At least 1 of the elements in the revised RE-AIM/PRISM framework reported as a study outcome. This can be quantitative (usually process outcomes) or qualitative	Models of all types (from simple scoring to machine-learning based models) and outcomes predicted (e.g., mortality, risk of ICU admission, etc) are included
Time	After implementation (for effectiveness); Before, during or after implementation (for other elements)	

All types of primary research studies were included (e.g., randomized, quasi-experimental, observational, qualitative, etc). For reviews that met our inclusion criteria, we included the relevant primary studies within instead of the review itself, allowing more flexibility with data extraction and synthesis.

We excluded conference abstracts and papers, theses and dissertations, letters to editors and commentaries as these were unlikely to contain sufficient information to contribute meaningfully to the review. We also excluded non-research documents in the grey literature and studies with no full-text in English due to lack of resources for searching and translation, respectively.

A wide variety of predictive tools may be used in the ED. For the sake of clarity, the following types of interventions were not included:

- Triage models that rely on subjective judgement and/or do not involve quantitative variables, including simple criteria-based rules.
- Models used in the ED but not applied on patients (e.g., for operations planning, staff roster planning, analysis of scans or reports, etc).
- Models for prediction or prognostication based on a single predictor or diagnostic procedure (e.g., troponin, CT scan, etc).
- Treatment protocols, guidelines, or pathways with multiple decision points/actions. Although some of these may include triage or prediction, they are typically only one of many components of a complex intervention. It is therefore difficult to assess the implementation aspects of the prediction component alone. We will focus on studies where the prediction model is the primary focus for implementation.
- Models or tools for diagnosis or measuring a single construct (e.g., pain, alcohol intake, etc).
- Models focused on improving operational efficiency (e.g., quality improvement studies).

### Information sources

We searched 4 electronic databases from the time of their inception until 30 June 2021: MEDLINE (through PubMed), Embase, Scopus and CINAHL. In addition, the reference lists of relevant reviews and articles included at the full-text screening stage will be screened for any additional studies.

### Search strategy

A list of keywords and index terms for informative PICOT elements for the intervention and implementation strategy was generated. The index terms for each database were searched and curated according to the controlled vocabulary of the database. For example, for PubMed, MeSH terms were searched using the keywords and the most relevant ones chosen. The keywords and index terms within each concept were then combined using the BOOLEAN operator ‘OR’ and searched in all databases. The results from the 3 concepts were then combined using the BOOLEAN operator ‘AND’ to narrow the search. The team then reviewed a sample of the initial search results and updated the search terms with additional keywords found in relevant articles. The following filters were applied: English, “Full text” and “Journal Article” to remove conference abstracts and other non-research articles. The final search terms are shown in Table 2.

**Table 2:** Literature search terms.

Concept	Population (intervention)	Intervention (intervention)	Outcome (implementation strategy)
	“emergency department” “emergency room”	“predictive score” “predictive model” “predictive rule” “prediction score” “prediction model” “prediction rule” “machine learning” “artificial intelligence” “early warning score” “triage”	Implement*
PubMed MeSH terms	Emergency Service, Hospital	“early warning score” “machine learning” “artificial intelligence” “triage”	“Implementation science”
Embase Emtree terms	“emergency ward”	“clinical decision rule” “machine learning” ‘artificial intelligence” “early warning score”	
CINAHL subject headings	“Emergency service”	“prediction models” “clinical prediction rules” “machine learning” “artificial intelligence” “early warning score” “triage”	“Implementation Science” “Program Implementation”

### Selection of sources of evidence

The entire team developed and piloted the search strategy. After the search strategy was finalized, the actual search was performed by one reviewer in all the databases (SLC). The results from searches in all databases were combined and duplicates removed using EndNote. The resultant list of citations was then imported into Rayyan.ai for screening [27]. In the first level of screening, two reviewers (SLC and JWL) screened the titles and abstracts independently and selected articles for full-text review. Any discrepancies were resolved by consensus with a third reviewer (NL). Next, two reviewers (SLC and JWL) screened the full-texts of articles selected for inclusion and any discrepancies were resolved by consensus with a third reviewer (NL). The reference lists of included articles were then scanned for further relevant articles. These additional articles were also subject to the same screening process as the initially included articles. The search will be repeated prior to writing up the results to capture any new articles that may be eligible.

### Data charting process

Two reviewers (SLC and JWL) will extract information from included articles using a charting form independently. The initial form may be revised to include additional relevant fields after the first 5 articles. Information extracted by both reviewers will then be combined and summarized. Any substantial discrepancies will be resolved by consensus with a third reviewer (NL).

### Data items

The initial variables that will be extracted are:

- Citation details (authors, title, year of publication, journal, volume, issue, pages)
- Country
- Context (institution name, type of hospital, type of setting, hospital size)
- Study design
- Study period
- Participants (description)
- Intervention details (model name, type, outcome(s), performance, validation status)
- Intervention strategy (actor, action, action target, temporality, dose)
- Methods (for each outcome, including sample size)
- Results
- Other information relevant to the RE-AIM/PRISM framework

### Synthesis of results

A description of included studies will be done in terms of the characteristics of the ED (country, context, participants), study design and model characteristics. Results and any information relevant to the RE-AIM/PRISM framework will be categorized according to the elements in the framework. The definitions of each outcome or domain given by the originators of the RE-AIM and PRISM frameworks, respectively, will be used to guide the categorization [28,29]. We will summarize the results by type of RE-AIM outcome(s) and PRISM domain by providing a descriptive summary (for quantitative outcomes, as appropriate) or narrative summary (for qualitative outcomes). The revised RE-AIM/PRISM also emphasizes fit among context, intervention and implementation strategies, and explicitly includes costs and adaptations under overarching issues. Where appropriate, we will also provide a narrative summary of these aspects [30]. Results may be presented by type of study, intervention, type of setting and presence, type or dose of implementation strategies, as appropriate. As ‘real-world effectiveness’ will be influenced by both the efficacy of the model (i.e., accuracy) and implementation success, we may also discuss the results in light of validation results and implementation strategies (if present) where appropriate.

## Discussion

This scoping review is the first to focus on the implementation process and real-world outcomes of prediction models implemented in the ED. The body of existing reviews on prediction models in the ED have principally focused on model development and validation [7,12,18]. This review aims to fill a gap in the current literature and complement existing reviews by providing an overview of how validated prediction models in routine clinical use are implemented and evaluated. It will therefore complement existing reviews that focus only on the performance of prediction models. Additionally, considering the burgeoning prevalence of high-performing Artificial Intelligence (AI) based models [31] and the increasing adoption of computerized clinical decision support systems [32], an inquiry into the implementation of prediction models carries tremendous practical implications. This is especially so in the ED, where a range of undesirable outcomes such as overcrowding [33], patient readmission [34] and septic shock [35] continue to persist even after patients leave the ED and are not directly addressed by triage itself.

Despite advancements in the development and validation of ED-based prediction models, there has been comparatively little progress in the implementation and integration of such models into clinical practice. One major barrier to adoption of prediction models is lack of evidence of clinical utility, which requires the model to provide information over and above what is already known, thereby prompting actions that lead to improved outcomes compared to without the model [36]. A host of other challenges such as data barriers, lack of transparency, regulation and certification, ethics, need for education and training, exists especially for models designed to harness electronic medical records to produce real-time prediction [37]. This review will summarize the collective experience of implementing various types of prediction models in EDs around the world using an implementation science framework and provide a sense of what factors promote or hinder implementation, what strategies might work in promoting implementation, how models perform in the real world and which area are lacking in implementation research. For EDs considering implementing certain prediction models in their setting, this review can provide valuable information on their model’s potential clinical impact, key strategies to maximize implementation success and what implementation studies to perform while doing so. This review can also reveal pitfalls and gaps in the implementation of certain prediction models. Taken together, a collection of both positive and negative real-world experiences can provide a holistic perspective of ED prediction model implementation, potentially aiding and streamlining the implementation process for future prediction models.

There are some strengths and limitations of our study. The key strength is that this review focuses on implementation, which is a gap in the review literature currently. Another strength is that we intend to include a broad range of models, which increases the applicability of the findings. The limitations are firstly, the exclusion of protocols, guidelines or pathways that include a prediction component. Prediction models are often not used in isolation but part of a care plan. However, the focus of this review is to inform how to implement new prediction models that are likely to be an addition to current clinical workflows rather than creation of a whole new workflow, although that might be necessary in some cases. Secondly, the summary of implementation outcomes may require our interpretation and categorization of study findings. This is inevitable and necessary as terminology within the implementation science is not standardized [38]. Moreover, many studies may not even have explicitly utilized implementation science methods or tools.

In conclusion, this scoping review will be a valuable resource for informing future implementation studies of prediction models in the ED.
